# Current Approach to Risk Factors and Biomarkers of Intestinal Fibrosis in Inflammatory Bowel Disease

**DOI:** 10.3390/medicina60020305

**Published:** 2024-02-10

**Authors:** Patrycja Dudek, Renata Talar-Wojnarowska

**Affiliations:** Department of Digestive Tract Diseases, Medical University of Lodz, 90-153 Lodz, Poland; dudek.patrycja.m@gmail.com

**Keywords:** IBD, strictures, fibrosis, biomarkers

## Abstract

Inflammatory bowel disease (IBD), especially Crohn’s disease (CD), characterized by a chronic inflammatory process and progressive intestinal tissue damage, leads to the unrestrained proliferation of mesenchymal cells and the development of bowel strictures. Complications induced by fibrosis are related to high rates of morbidity and mortality and lead to a substantial number of hospitalizations and surgical procedures, generating high healthcare costs. The development of easily obtained, reliable fibrogenesis biomarkers is essential to provide an important complementary tool to existing diagnostic and prognostic methods in IBD management, guiding decisions on the intensification of pharmacotherapy, proceeding to surgical methods of treatment and monitoring the efficacy of anti-fibrotic therapy in the future. The most promising potential markers of fibrosis include cartilage oligomeric matrix protein (COMP), hepatocyte growth factor activator (HGFA), and fibronectin isoform- extra domain A (ED-A), as well as antibodies against granulocyte macrophage colony-stimulating factor (GM-CSF Ab), cathelicidin (LL-37), or circulatory miRNAs: miR-19a-3p and miR-19b-3p. This review summarizes the role of genetic predisposition, and risk factors and serological markers potentially contributing to the pathophysiology of fibrotic strictures in the course of IBD.

## 1. Introduction

Inflammatory bowel disease (IBD), such as Crohn’s disease (CD) and ulcerative colitis (UC), is characterized by a persistent state of inflammation and progressive intestinal tissue damage, which may lead to uncontrollable mesenchymal cells proliferation and the accumulation of an excessive amount of extracellular matrix (ECM) ingredients. These processes contribute to bowel wall thickening, the development of strictures, and subsequently obstruction—one of the most common complications in the course of IBD, especially CD. The behavior and natural course of CD is highly heterogenous, while the location of the disease remains relatively stable [1,2,3]. According to the Vienna classification, at the moment of diagnosis, 77% of CD patients were categorized as having the pure inflammatory phenotype of the disease, whereas the development of strictures and fistulae was noticed in 11% and 16% of patients, respectively [3]. This pattern changes dramatically over time, and 5 years after diagnosis, complication rates in patients with CD were reported to range between 48 and 52%. Moreover, 10 years after diagnosis, complications occurred in up to 70% of CD patients, with approximately half of them developing strictures [2,3,4]. The risk of needing surgical treatment among CD patients is estimated to be between 40 and 71% in the 10-year period after diagnosis [5,6]. The main indications for surgical proceeding include strictures, abscesses, and fistulae. Most often, stricturing CD is treated with strictureplasty or surgical resection. However, recrudescence of the disease at an anastomosis site is frequent, with up to 73% of patients developing recurrent strictures 10 years after strictureplasty [7].

The localization with the greatest likelihood of forming de novo strictures is the ileum and the ileocolonic region. Probably, it is caused by the relatively smaller diameter of this part of the gastrointestinal (GI) tract. Nevertheless, stenotic complications may appear in any region of the GI tract affected by CD: the upper part of the GI tract, the colon, or the rectum. The frequency of stricture formation indicates the most common inflammation sites in the GI tract, with a number of 40–55% stenotic complications occurring in the terminal ileum and colon, 15–25% only in the colon, 25–40% in the ileum alone, and up to 10% affecting the upper part of the GI tract [8,9].

UC, the second main type of IBD, is manifested by continuous inflammatory lesions affecting the inner lining of the large bowel. In the past, UC has not been related to the process of fibrosis. However, recent studies have shown the presence of submucosal fibrosis in up to 100% of colectomy samples from UC patients qualified for surgical treatment due to dysplasia, cancer, or refractory disease [10]. The fibrosis rate is relative to the degree of chronic, but not active inflammation [10,11]. Compared to CD, strictures in UC are much less frequent due to the location of the disease being limited to the large bowel and a wide lumen of the colon. In this form of IBD, stricture formation ranges from 1 to 11.2% of UC patients [12]. Individuals developing strictures should always undergo oncological screening, as a significant proportion of these complications may be related to colorectal cancer. In order to prevent malignant transformation and fibrostenotic complications in UC patients, it is recommended to introduce early colonoscopy surveillance and active anti-inflammatory treatment for better control of the course of the disease [13].

Stricture formation among IBD patients leads to a growing number of hospitalizations, often including surgical treatment, generating high healthcare costs and considerably reducing the quality of life of affected individuals. Easily obtained, reliable biomarkers, such as blood-based markers, would be an essential, complementary instrument in diagnosis, therapy, and monitoring the course of IBD.

This paper aims to summarize the risk factors and biomarkers potentially contributing to the pathophysiology of fibrotic strictures in the course of IBD. The role of genetic predisposition in the development of stenotic complications will also be discussed.

## 2. Pathophysiology of Intestinal Fibrosis

**Figure 1:**
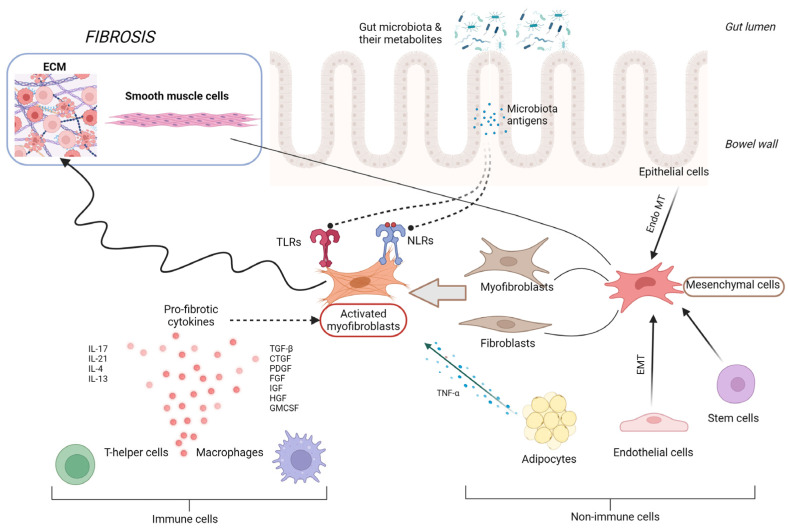
Pathophysiology of intestinal fibrosis. CTGF, connective tissue growth factor; ECM, extracellular matrix; EMT, epithelial–mesenchymal transition; Endo MT, endothelial–mesenchymal transition; FGF, fibroblast growth factor; GMCSF, granulocyte-macrophage colony-stimulating factor; HGF, hepatocyte growth factor; IGF, insulin-like growth factor; IL, interleukin; NLRs, NOD-like receptors; PDGF, platelet derived growth factor; TGF-β, transforming growth factor β; and TNF-α, tumor necrosis factor α.

Intestinal fibrogenesis is a complex, multifactorial process affected by multiple elements, such as genetic factors, gut barrier integrity, microbiota, the immune system, or the regulation of cytokine expression (Figure 1). Two parallel processes are responsible for fibrogenesis in IBD: the expansion of smooth muscle cells and the extensive accumulation of ECM in layers of the bowel wall [14]. In the situation of intestinal tissue damage, the process of mesenchymal cells’ accumulation starts in order to secrete ECM components together with growth factors and repair the defect. Mesenchymal cells, characterized by a high motility and versatility, may be gained in the process of the proliferation of existing local mesenchymal cells, cell migration from adherent structures, or differentiation from other types of intestinal cells, like epithelial or endothelial [15]. Intestinal microorganisms and their metabolites, together with growth factors, cytokines secreted by immune and non-immune cells, or even ECM products themselves, are the main factors inducing the processes of mesenchymal cells’ activation and differentiation [16]. One of the potential targets of several triggering factors, especially microbial components, are toll-like receptors (TLRs), mainly TLR-4, the activity of which affects epithelial–mesenchymal transition, collagen production, or myofibroblast function [17]. However, in a situation of chronic, severe inflammation, like in IBD, mechanisms of tissue self-regeneration become upregulated, resulting in the accumulation of excessive amounts of ECM products, reducing the intestinal lumen in the place of previous injury, developing stenosis, and subsequently GI tract obstruction. The chronically stimulated mechanisms of tissue regeneration lead to an imbalance between the matrix metalloproteinases (MMP) and cathepsins involved in tissue degradation and the tissue inhibitors of metalloproteinases (TIMPs), impeding their activity [18,19]. The progression of fibrotic changes in the bowel wall can continue independently from the activity of inflammation, which seems to be only a triggering factor for the onset of fibrosis, proceeding in its next steps in a self-perpetuating manner, activated by integrin-mediated mechanisms [19,20].

One of the latest findings in the field of ileal fibrosis pathogenesis applies to the role of gut microbiota reactive antigen-specific T helper (Th) 17 cells. The study by Zhao et al., performed on a mouse and human model, showed that Th17 cells induce intestinal fibrosis via the expression of the epidermal growth factor receptor ligand amphiregulin (AREG) [21]. The intestinal CD4+ T cells of CD patients presented augmented AREG expression in fibrotic sites compared with nonfibrotic bowel segments. Hence, AREG may serve as a new potential biomarker of fibrosis and target for anti-fibrotic treatment in the future. Furthermore, the study proved that, despite multiple analyses of ileal fibrosis pathomechanisms, our knowledge about these processes is still deficient.

A more detailed depiction of the mechanisms involved in the process of intestinal fibrogenesis is beyond the scope of this review and has been raised in other publications.

## 3. Risk Factors of Fibrogenesis

### 3.1. Clinical and Environmental Risk Factors

The most commonly studied risk factors of the fibrostenotic CD course include clinical and endoscopic parameters. Nevertheless, it has to be noted that most of the discussed factors are not specific predictors of the fibrostenotising phenotype of the disease, but should be rather considered as parameters showing a tendency towards developing a more serious IBD course, including stricture formation. The risk of the stricturing CD course seems to be independent of sex [22,23]. Its clinical parameters, mostly used for predicting a more complicated phenotype of CD, are: small bowel disease location, perianal disease at diagnosis, and an initial requirement for steroids use [22,24,25]. Most studies also mention a young age at diagnosis as being a risk factor for a complicated CD course, generally defined as the onset of the disease <40 years of age [22]. However, a retrospective cohort study conducted on 1936 IBD patients showed contradictory results. The risk of surgery due to stricturing complications was increased in patients with CD who were 45–59 years of age at diagnosis (*p* = 0.0023) compared to those aged 15–29 years at diagnosis [23]. Some studies also reported the need for early azathioprine (AZA) therapy as a predictor of disease behavior changes in CD patients. A study conducted on a cohort of 340 CD patients showed that the early use of AZA (*p* = 0.005), as well as AZA/biological therapy (*p* = 0.002), was associated with disease behavior changes from B1 (inflammatory phenotype) to B2 (stricturing phenotype)/B3 (penetrating phenotype) [24].

A history of smoking is an environmental risk factor also considered to be of great importance for a more complicated CD course and more rapid progression from diagnosis to the formation of the first stricture. Some previous studies have suggested smoking to be associated with a greater probability of progression to a complicated phenotype of the disease, meaning the development of strictures or fistulae [25,26,27,28]. According to other authors, the risk of surgical treatment and further resections during the disease course tends to be higher among smoking individuals [29]. Cosnes et al. found steroids and immunosuppressants requirement to be higher in smokers compared with non-smokers [30]. The mechanisms of the effect of smoking on IBD course are not clear. Most data come from past studies performed in the 1980s and 1990s. Components of tobacco smoke, like nicotine or carbon monoxide, lead to an immunosuppressive effect of smoking, influencing both cellular and humoral immunity. They alter immunoglobulin (Ig) levels, reducing the concentration of serum IgG [31]. They may also change the proportion of immunoregulatory T cells, inducing a reduction in the ratio of T-helper to T-suppressor cells [32]. Smoking has also been connected with altering mucus secretion and the composition in the bowel lumen, what may influence the gut barrier integrity [33]. Furthermore, it may enhance the dysfunction of ileal microvascular perfusion [34].

### 3.2. Endoscopic Risk Factors

Endoscopy techniques are sensitive methods for the investigation of changes in the superficial layers of the GI tract. However, these procedures are able only to detect severe narrowing of the lumen by visualization or an inability to pass the endoscope and are not appropriate for assessing transmural changes. Endoscopy results can be only partially related to the prediction of a stricturing phenotype, as they rather reflect the activity of the disease and show the IBD behavior well after some complications have occurred. However, some endoscopic findings, such as disease location or mucosal lesions, are considered as predictors of an aggressive disease course. The risk of surgical intervention tends to be higher among patients with extensive, deep, and active mucosal ulcerations [35]. A retrospective study performed by Allez et al. suggested that CD patients with a higher risk of surgery and penetrating complications have a more aggressive course of the disease, with severe lesions in the ileocolon being visualized during endoscopy at symptomatic phases. In a group of 102 patients included in the study, 53 were identified with severe lesions at index colonoscopy, defined as extensive, deep ulcerations affecting more than 10% of the mucosa of a minimum of one colonic segment. During the median 52 months of follow-up, 37 individuals underwent colectomy. The authors observed that the colectomy rate was significantly higher among patients with severe endoscopic lesions compared with those without severe lesions [36].

Disease site is also associated with a complicated course of CD and the need for surgery [37]. The small bowel location of inflammatory changes, rather than the colon, has been defined as being predictive of progression towards stricturing disease and a higher rate of surgery [25]. According to Louis et al., the ileal location of CD is linked with a stricturing phenotype, whereas frequent exacerbations are associated with a penetrating phenotype. The study was performed on a total of 163 CD patients with a non-penetrating, non-stricturing pattern at diagnosis [25]. These conclusions were confirmed in a study by Lakatos et al. performed on 344 CD patients. The results suggest that disease location in the small intestine (*p* = 0.001) and the recognition of perianal disease (*p* < 0.001) are independent predictors of disease behavior changes in CD patients [24]. It becomes evident that groupings of the disease, in particular the Montreal classification, merely identify fibrotic changes after they have become clinically significant. Using this classification to assess risk factors seems to have substantial limitations [38].

On the contrary to CD, the relative risk of stricture formation in UC is much lower. One of the known risk factors of fibrostenosis in UC is the duration of the disease [39,40]. In the study published by Yamagata et al., disease duration was identified as being significantly longer in UC patients with stricturing disease (15.6 years) compared to those without strictures (8.6 years). In this cohort, the incidence of benign stenosis was rated 1.5% over 23 years [40]. Gordon et al. observed a significant association between submucosal fibrosis and the severity of intestinal inflammation (*p* < 0.001), as well as histopathological changes in chronic mucosal injury. No correlation with active inflammation was found. Furthermore, there were no features found on endoscopic mucosal biopsies able to assess the size of the underlying fibrosis or the thickness of the muscularis mucosae [10]. A study conducted on a pediatric population with UC confirmed this hypothesis. In the pediatric UC patients, colorectal submucosal fibrosis and the thickening of the muscularis mucosa were correlated with the presence, chronicity, and degree of inflammation of the mucosa [41]. However, a significant proportion of stenotic complications have been related to the presence of cancer [12,40]. In the study conducted on 1156 patients with UC, 59 of them had colon stenosis, with 24% of these patients being diagnosed with colorectal cancer [12]. The risk of developing colorectal cancer was associated with the duration of the disease (>20 years), location of the disease proximal to the splenic flexure of the colon, and the symptomatic course of stenosis formation. Additionally, the risk of malignant stenotic changes is increased in patients with extensive, active inflammation involving a large part of the intestine, with primary sclerosing cholangitis, or a family history of colorectal cancer < 50 years of age [13].

### 3.3. Imaging Techniques in Fibrostenosis Evaluation

Apart from endoscopy techniques, there are several radiological modalities used for the assessment of IBD complications, including fibrostenosis. In the face of a lack of reliable, clinically useful laboratory markers, radiological techniques are still crucial in the process of assessing fibrostenotic changes in the intestinal tract. For many years, the main problem in using imaging modalities in the diagnosis of fibrostenotic complications referred to a lack of standardized definitions for GI tract strictures. A group of international IBD experts—the CrOhN’s disease anti-fibrotic STRICTure therapies (CONSTRICT) group—has provided some defined radiological criteria for ileal stenosis. Due to consensus, a naïve small bowel stricture may be defined as a combination of three features found in cross-sectional imaging: localized luminal narrowing (reduction in luminal diameter of at least 50%, compared to the adjacent normal bowel tract), bowel wall thickening (25% increase in wall thickness compared to the adjacent healthy bowel loop), and pre-stricture dilation (luminal diameter more than 3 cm) [42]. All available cross-sectional imaging techniques today, like Computed Tomography (CT), Magnetic Resonance (MR), and Intestinal Ultrasound (IUS), have the ability to detect strictures, varying in terms of accuracy, availability, exposure to radiation, or cost effectiveness. One of the greatest concerns remains distinguishing between inflammatory-predominant strictures and the fibrotic type of bowel stenosis, as none of the currently available imaging techniques are able to accurately assess the amount of accumulated fibrosis.

CT techniques, including CT enterography (CTE), are characterized by a high sensitivity and specificity in bowel stenosis identification, reaching 85–100% and up to 100%, respectively [43,44,45]. The main limitation of this type of testing refers to radiation exposure, which may exclude from its use a significant group of CD patients—the pediatric population or young adults, who are especially susceptible to the long-term effects of radiation. Another limitation includes the questionable clinical usefulness of CTE in distinguishing different types of strictures and identifying those associated with fibrostenosis. A retrospective study conducted on a group of 22 CD patients, who underwent a surgical resection of a small bowel stricture, showed that CTE was a sensitive tool for identifying inflammatory changes (*p* = 0.002), such as mesenteric hypervascularity, mesenteric fat stranding, and mucosal hyperenhancement; however, it did not predict the presence of tissue fibrosis [46].

MR modalities, especially MR enterography (MRE), have gained popularity in CD management in the last years. MRE is characterized by being comparable to CTE sensitivity and specificity in stenosis identification, estimated at 75–92% and 90–95%, respectively [47,48]. However, the main advantage of MR modalities is the lack of exposure to radiation, altogether making MR an optimal technique for the diagnosis of intestinal strictures and assessment of anti-fibrotic therapy response [42]. Limitations of this technique include restricted availability, a long examination time, and higher costs, in comparison to CT. MR findings predictive for stenosis include T1 and T2 isointensity or hypointensity, delayed mural hyperenhancement relative to the normal bowel, and an elevated magnetization transfer ratio [49]. Recently, some novel modalities of MR imaging have been tested, such as Type I Collagen Targeted MR Imaging Probe. A study held on a rat model showed a correlation with the severity of bowel fibrosis (*p* = 0.021), presenting this technique as a promising method for predicting the progression of fibrotic changes and monitoring the therapeutic response [50].

There are several ultrasound (US) techniques with high diagnostic potential in CD management and fibrosis detection: B-Mode IUS (B-IUS), strain elastography (SE), shear wave elastography (SWE), colour Doppler imaging (CDI) and contrast-enhanced ultrasound (CEUS). Markers of fibrostenosis in US include a thickened bowel wall with a lack of vascularity or contrast enhancement, prestenotic lumen dilation with an increased fluid content, and the presence of stratification in contrast with a loss of stratification typical for inflammatory changes with a low degree of fibrosis [49,51].

Due to its high availability, good tolerance among patients, and low costs, IUS with its modalities seems to be an ideal diagnostic tool for CD patients. However, recently published data have shown that US’s diagnostic value remains unclear [52]. The main limitation of IUS may be its high dependence on the skills and experience of the operator, which leads to significant variability in results. Furthermore, US techniques have a low ability to obtain some segments of GI tract, like the duodenum and rectum, as well presenting limited visualization among obese patients. Despite the great potential of these techniques, more studies are needed to understand the precise significance of each radiological parameter and assess cut-off values in different US modes. There is a high discrepancy in evaluating the diagnostic accuracy of US techniques in stenosis detection, with sensitivity varying from 74% to 100% and specificity ranging from 89% to 91% [53,54,55]. The detection of stenosis improves significantly when using a US modality with contrast enhancement. In a study comparing the accuracy of conventional US and contrast-enhanced techniques in assessing CD complications, i.a., intestinal stenosis, the sensitivity in stricture detection was 74% and 89% for conventional and contrast US, respectively [56]. In another study, the diagnostic value of transabdominal US and contrast US in small bowel lesion detection was evaluated on a group of 28 CD patients. The sensitivity of at least one stricture detection was 76% for conventional US and 94% for the contrast-enhanced technique [57].

A great challenge in CD stricture diagnosis rises from distinguishing inflammatory from fibrostenotic lesions, what may be a matter of great importance in CD management therapy choice, treatment modification, or shifting to surgical procedures. US techniques, such as CDI or CEUS, seem to be a promising tool for the differentiation of such lesions, as they are able to assess the parameters of the bowel wall, appropriate for evaluating the grade of fibrosis in the thickened wall, vascularity, perfusion, neoangiogenesis, and the presence of piercing vessels. However, available data on the clinical usefulness of US modalities in assessing fibrosis in bowel strictures are scarce. A recently published meta-analysis including 14 studies showed that US techniques were inaccurate in differentiating inflammatory from fibrotic stenosis [52]. Another US technique—elastography—also seems to be a promising tool in assessing fibrostenosis in CD patients. Its clinical usefulness is based on changes in the mechanical and elastic properties of the bowel wall due to ECM products’ deposition and smooth muscle proliferation in the process of fibrosis, making the measurement of ileal tissue stiffness a marker of fibrosis [58]. Elastography includes two modalities: strain elastography (SE), which measures the bowel stiffness in response to external tissue compression, and shear wave elastography (SWE), the function of which is based on the speed of acoustic wave propagation in tissues differing in stiffness. In the study by Fraquelli et al. conducted on 23 CD patients qualified for terminal ileum resection, SE strain ratio measurements correlated significantly with the severity of fibrotic bowel lesions in a histological image analysis (*p* < 0.0001) [59]. In another study, authors assessed the use of real-time elastography (RTE) in bowel fibrosis detection. Affected and unaffected by stenotic changes, the ileal segments of 10 CD patients were examined pre-, intra-, and postoperatively with different techniques, with a correlation found between RTE, direct tensiometry, and the histological examination results [60]. Further studies evaluating the clinical usefulness of elastography are needed due to small study groups, the high heterogeneity of the used modalities, and no established cut-off values, which hampers defining the role of these techniques in distinguishing different types of bowel stenosis.

### 3.4. Biomarkers of Fibrosis in IBD

Multiple studies have tried to identify laboratory parameters and biomarkers which would be able to estimate the risk of the fibrostenotic course of IBD, detect the initial stages of fibrosis prior to symptoms, and assess the outcome of a patient’s therapy. Table 1 summarizes the identified potential biomarkers—serologic, genetic, and histologic—associated with stenotic complications.

Comparably, little attention has been focused on routinely used and widely available tests, such as C-reactive protein (CRP) concentration. Multiple studies have evaluated the use of CRP in IBD, especially in CD, for establishing a diagnosis, monitoring disease activity, or assessing the response to treatment. The role of CRP as a predictive biomarker in GI tract stricture formation is unclear and study results remain inconsistent. In a cross-sectional study using proteomics to identify potential biomarkers of stricturing CD, no significant correlation with CRP, leukocyte, platelet, and hemoglobin concentration was found [61]. A newly published study demonstrated that a higher serum erythrocyte sedimentation rate and platelet counts, but not CRP, were associated with CD patients’ strictures [62].

### 3.5. Extracellular Matrix Proteins

Using ECM proteins as potential biomarkers of intestinal fibrogenesis intuitively seems to be an expected proceeding, as an excessive accumulation of ECM products is related to the process of remodeling and stricture formation in the intestinal wall. The predominant matrix molecules are collagens, with two major types—collagen I and collagen III—being involved in fibrogenesis [63]. Several studies have evaluated the circulating metabolites of connective tissue, but the results were not consistent and collagens and their properties did not receive the status of being a reliable biomarker of intestinal fibrostenosis [63,64]. More promising outcomes were achieved in a study based on the measurement of the serum levels of molecules involved in collagen turnover and degradation (fragments of collagen type I (C1M), III (PRO-C3, C3M), IV (PRO-C4, C4M, C4G), and VI (C6Ma3)) in a group of CD patients in comparison to healthy individuals. A high level of degradation of collagen type I, III, and IV and excessive formation of collagen type IV were associated with the stricturing phenotype of CD [65].

Recently, some interesting findings were also reported concerning fibronectin. Fibronectin can occur in up to 20 different isoforms due to the alternative splicing of the primary transcript, with every isoform having a different function. Splicing variant ED-A is connected to cell proliferation and the differentiation of fibroblasts into myofibroblasts. Tissue stiffness is one of the known factors which affects the alternative splicing of fibronectin. In the study by de Bruyn et al., increased expression of fibronectin isoform ED-A was observed in an immunohistochemical examination of intestinal samples obtained from CD patients unresponsive to infliximab (IFX) therapy, who underwent ileocecal resection. According to this study, the tissue of the IFX failure patients was characterized by increased stiffness because of higher levels of collagen and fibronectin. The thickness of the muscularis mucosa of those individuals was substantially greater than the mucosa of subjects naïve to IFX [66].

Cartilage oligomeric matrix protein (COMP) is a glycoprotein from the thrombospondin family, which takes part in ECM production and tissue remodeling in response to damage [67]. COMP interacts with other ECM components, including different types of collagen (I, II, IX, XII, and XIV), matrillin-3, aggrecan, fibronectin, and proteases (MMP-3,-12,-13), directly linked to ECM formation [68]. Other roles of COMP include ECM protein export and the correct integration of ECM. Disorders of these functions cause skeletal dysplasias, wound healing abnormalities, and fibrosis in multiple organ systems [69,70]. The function of COMP is highly integrated with transforming growth factor β (TGF-β), which plays an important role in regulating myofibroblast activity and ECM characteristics. In the process of fibrosis, COMP and TGF-β interact mutually, affecting the activity and expression of each other, in a self-perpetuating cycle [71,72]. A dysregulated expression of COMP has been found in numerous pathologies connected to cartilage destruction and fibrosis, like rheumathoid arthritis, idiopathic pulmonary fibrosis, or scleroderma [72,73,74]. The clinical potential of COMP as a biomarker is associated with the secretion of high levels of this protein into the bloodstream, which enables an indication of COMP serum concentration using conventional methods. In a study conducted by Stidham et al., subjects with fibrostenotic and inflammation-predominant CD phenotypes underwent a comparison of their quantitative serum glycoproteome profiles [61]. The COMP serum levels were elevated in the fibrostenotic vs. inlammatory CD group of patients (*p* = 0.012). Increased concentrations of COMP among subjects with fibrostenosis persisted even after the resection of the affected parts of the intestine. The constantly elevated COMP expression may exhibit a susceptibility for fibrotic changes in response to tissue damage and inflammation.

### 3.6. Growth Factors

Another group of interest as potential biomarkers of fibrostenosis are growth factors. Among these, transforming growth factor β (TGF-β) plays a predominant role, regulating the process of fibrosis in many organs, including the intestine, contributing to disorders such as diabetic nephropathy, rheumathoid arthritis, radiation-induced fibrosis, or myocarditis [75,76,77,78,79]. TGF-β belongs to a large superfamily of activins/bone morphogenetic proteins. Produced by various types of cells, TGF-β is characterized by pleiotropic activity, including the regulation of the immune response, cell proliferation, and oncogenesis. The association between TGF-β level and intestinal strictures in CD patients was investigated, and it was proved that the expression of TGF-β was increased in the intestinal mucosa covering strictures compared to non-strictured parts of the intestines of patients with fibrostenosing CD [80]. An elevated level of expression of TGF-β1 and active TGF-β1 was also found in the muscle cells of intestinal strictures, obtained from surgically resected ileal segments of CD patients [81].

Hepatocyte growth factor activator (HGFA) is a protease secreted into the blood by the liver in order to activate hepatocyte growth factor (HGF) as a response to tissue damage. HGF is a multipotent molecule produced by various types of cells, including fibroblasts, taking part in crucial processes such as the regeneration and protection of tissues, epithelial to mesenchymal cell transformation, the apoptosis of myofibroblasts or protection from chronic inflammation, and fibrosis [82]. HGF has an antagonistic relationship with TGF-β, inhibiting fibrotic remodeling [83]. The administration of HGF or HGF gene therapy contributes to anti-fibrotic effects in lung, liver, renal, cardiac, and brain injuries, which was confirmed in animal models [84,85,86,87,88]. In the aforementioned study by Stidham et al., HGFA serum levels were significantly elevated in a fibrostenotic group of CD patients compared to subjects with the inflammatory phenotype (*p* = 0.031). Within the group with the fibrosis-predominant phenotype, HGFA levels significantly declined following the resection of the fibrostenotic intestine (*p* = 0.015). Elevated serum HGFA levels in fibrostenotic subjects, with a significant decline after surgical resection, suggest the usefulness of this enzyme as a marker of accumulated fibrotic bowel damage [61].

### 3.7. Cytokine Antibodies

Endogenous autoantibodies to cytokines are able to modulate inflammation by creating a state of relative immunodeficiency in IBD patients, predisposing them to chronic inflammatory processes in the intestinal mucosa. In the study by Ebert et al., the concentration of antibodies recognizing TGF-β was significantly higher in UC patients (*p* < 0.01), compared with normal sera. In the same study, anti-IL-10 antibody levels were found to be greater in CD (*p* < 0.05) patients than among healthy individuals [89]. In a subsequent study, an increased concentration of neutralizing autoantibodies against granulocyte macrophage colony-stimulating factor (GM-CSF Ab) was observed in a population of adult and pediatric CD patients, however, GM-CSF Ab level was found to be especially elevated among subjects with ileal disease involvement and the stricturing CD phenotype (*p* < 0.001). Another important finding in this research, performed additionally on an animal model, was the loss of the barrier function of the intestinal mucosa and the development of transmural ileitis after exposure to non-steroidal anti-inflammatory drugs (NSAID) in GM-CSF-null mice and NOD2-null mice, in which GM-CSF was neutralized [90]. Parallel results were obtained in another study. The authors found that an elevated concentration of GM-CSF Ab, disease duration greater than 3 years, and ileal location of the disease were independent risk factors of stricturing/penetrating CD behavior and intestinal resection [91]. GM-CSF, which is produced by the immune cells of the lamina propria, plays an important role in regulating intestinal inflammatory processes by supporting epithelial barrier integrity or stimulating crypt cell proliferation in acute tissue injury. Deficiency of GM-CSF can contribute to a relative immunodeficiency and disorder in ileal homeostasis [92].

### 3.8. Antimicrobial Antibodies

Searching for biomarkers associated with the gut microbiota, such as antimicrobial antibodies or antimicrobial proteins, seems to be promising, as molecules connected to enteric flora might be unique markers specific for intestinal fibrosis, distinguishing ileal from other types of organ fibrosis. Dysbiosis is related to IBD in general, and in addition, changes in enteric microbiota composition may be characteristic for different types of disease phenotypes [93]. Alterations in the gut microbiota and their metabolites, together with a loss of ileal barrier integrity, lead to the translocation of microbial antigens to the bowel mucosa or portal circulation and indirectly stimulate the production of fibrotic agents by immune and non-immune cells [94]. The process of antigen recognition by immune and non-immune cells takes place with a contribution from pattern recognition receptors (PRRs), like Toll-like receptors (TLRs) and nucleotide-binding and oligomerization domain (NOD)-like receptors (NLRs) [95]. Due to the role of dysregulated intestinal immune response in the pathogenesis of IBD, multiple studies have been conducted evaluating the clinical usefulness of antimicrobial antibodies in UC and, in particular, CD management [96]. An association between antimicrobial antibodies level and IBD behavior or phenotype was the most prominent regarding anti-Saccharomyces cerevisiae antibodies (ASCA). A prospective cohort study evaluated their prevalence and relationship with IBD. Positive ASCA was found to occur more frequently in CD patients with stricturing (*p* = 0.003) or penetrating (*p* = 0.012) complications compared to subjects with the pure inflammatory phenotype of CD at diagnosis. Furthermore, patients with ASCA presence had at least a twice higher risk of the evolution of their disease course to being more severe during follow-up (*p* < 0.001) [97]. This association was confirmed in several other studies [96,97,98]. In the study by Degenhardt et al., ASCA IgG and IgA were qualitatively and quantitatively associated with CD, CD complications (fistula and stenosis), and the need for surgical treatment [98]. This research has also shown link between another antibody type—serum anti-zymogen granule membrane glycoprotein 2 (GP2) antibodies. GP2 is thought to play an important role in immunomodulatory processes. The expression of GP2 in human enterocytes suggests that the pathogenesis of Crohn’s disease is, apart from multiple other factors, associated with anti-GP2 response [99]. Anti-GP2 IgA and IgG levels were found to be exclusively connected to the stricturing CD course and the need for surgical intervention, independently of disease location. No significant association with the fistulizing phenotype of CD, early disease onset, or disease activity was found [98]. In another study, the results showed that CD patients with the presence of IgA and/or IgG ASCA antibodies and anti-GP2 IgG antibodies, compared to seronegative individuals, had an early disease onset (*p* < 0.0001) and greater risk of both ileal and colonic disease (*p* < 0.0001), as well as forming strictures (*p* < 0.0001) [100].

Other antibodies which are associated with the fibrostenotic CD phenotype include both antimicrobial molecules: anti-flagellins A4-Fla2, anti Fla-X, anti-CBir1, and anti- Escherichia coli outer membrane porin C (anti-OmpC), and those that are nonantimicrobial, such as antineutrophil cytoplasmic antibody (ANCA)/perinuclear ANCA (pANCA) [101,102,103,104,105]. Another antimicrobial peptide which gave promising results is cathelicidin (LL-37, also known as hCAP18). Cathelicidin expression was found in multiple tissues, like the mucosa of the colon, breast, salivary glands, or some types of immune cells. Cathelicidin in the intestinal epithelium is responsible for ensuring epithelial barrier integrity or bacterial adhesion. An increased expression of cathelicidin was found in the mucosa of UC patients [106]. A study performed by Tran et al. showed that serum cathelicidin levels were inversely correlated with the activity of the disease (Partial Mayo Score) in UC patients, which is consistent with its known anti-inflammatory effect. Cathelicidin concentration combined with CRP level indicated the activity of UC more accurately than using either of these parameters independently. The study also demonstrated that low LL-37 levels among CD patients indicated a higher risk of developing intestinal strictures (*p* = 0.0485); however, it was not determined whether LL-37 levels were associated with the development of other complications like fistulae. The study was performed on two cohorts of IBD patients—80 UC patients and 95 CD patients. The serum levels of LL-37 were assessed using ELISA tests [107].

### 3.9. Fecal Biomarkers

Fecal calprotectin (FC) and fecal lactoferrin (FL)-neutrophil-derived proteins are the two most commonly used fecal biomarkers in clinical trials. The role of FC is well-established, with a significant correlation with intestinal inflammation, serving as a useful tool in CD evaluation. The use of FL testing has been limited mainly to research, probably due to the low stability of lactoferrin at room temperature.

Although fecal biomarkers seem to have a great potential to serve as easily obtained, non-invasive indicators of structuring CD, available data concerning this type of markers are limited. Only a few studies have evaluated fecal markers in the context of GI tract strictures. In a recent study, FC and FL levels were assessed to predict disease recurrence in CD patients with anastomotic strictures who underwent surgical treatment. The patients included in the study were evaluated by postoperative colonoscopy. Endoscopic balloon dilation was performed in subjects with strictures at the site of anastomosis, unable to pass by the colonoscope, regardless of the patients’ symptoms. Stool samples for FC and FL were collected on the day preceding bowel cleaning. Both FC and FL levels were significantly associated with the endoscopic recurrence of anastomotic strictures (*p* < 0.001), with an optimal cut-off value of 90.85 μg/g for FC and 5.6 μg/g for FL [108]. The use of FC as a potential biomarker of stricturing CD was also discussed in a recently published article. The authors assessed the management of stricturing CD in two pregnant patients using FC levels and intestinal ultrasound, proving that FC can serve as a complementary tool to ultrasound findings in confirming therapeutic response. Moreover, an increased FC level during pregnancy is associated with later exacerbation and a higher risk of adverse fetal and maternal outcomes [109].

### 3.10. Tissue-Based Biomarkers

Histopathologic analyses of intestinal fibrosis may provide some critical information about the pathogenesis of stricture formation, leading to the development of antifibrotic therapies. The most remarkable changes in strictured intestinal tissue include chronic inflammation, hypertrophia of muscularis propria, and hyperplasia of the smooth muscle layer in the submucosa. The ‘inflammation–smooth muscle hyperplasia axis’ appears to be the crucial patomechanism of stricture formation in the course of CD [110]. However, no standardized scoring system to grade the severity of histological fibrosis is currently available, which hampers further investigations and comparisons of study results [111]. The main limitation to the clinical use of tissue-based biomarkers is their low availability, requiring endoscopic procedures, which makes them less significant in IBD management compared to easily obtained serum biomarkers. The second major objection is the limited value of endoscopic mucosa biopsy samples for diagnosis of intestinal fibrosis, as stricture formation is a transmural process. This may be the cause of lacking studies confirming histopathological biomarkers.

Potential markers include transforming growth factor beta activated kinase 1 (TAK1). A study conducted on 26 IBD patients evaluated surgical ileal samples obtained from individuals with the stricturing phenotype of CD. The concentrations of TAK1 and its phosphorylated form—pTAK1—were elevated in the ileal specimens of CD patients compared with healthy subjects and correlated with the level of intestinal fibrosis (*p* < 0.01) [112]. Another study evaluated the expression of fibrosis markers and pH-sensing receptors in ileal samples from CD patients who had undergone ileocaecal resection because of fibrostenotic complications. The expression of pH-sensing ovarian cancer G-protein coupled receptor-1 [OGR1/GPR68] was found to be elevated in the ileal samples of fibrostenotic patients and positively correlated with the expression of pro-fibrotic cytokines and pro-collagens (*p* = 0.016) [113]. In a different study, the authors observed a gradual increase in cholesterol 25 hydroxylase (CH25H) expression in samples, comparing, as follows: healthy control ileal tissue, non-fibrotic ileal tissue of CD patients, and fibrotic ileal tissues from the same CD patients (*p* < 0.05). Samples were obtained from subjects who underwent ileocaecal resection because of stenotic complications. The expression of CH25H was strongly correlated with the expressions of various fibrosis mediators (COL-1, COL-3, SMA, and TGF-β) [114]. As all the experiments were conducted on a small group of subjects, further studies are required to elucidate the exact significance of tissue-based markers.

### 3.11. Genetic Variants

Genetic variants have also been considered as markers of stricture formation in IBD. The first gene proven to be linked with CD was the nucleotide binding and oligomerization domain, named later the caspase activation recruitment domain (NOD2/CARD15). Later research proved the association of this gene with susceptibility to the stricturing phenotype of CD. A meta-analysis including CD patients showed that owners of at least one high-risk variant of NOD2/CARD15 had a slightly increased risk of familiar disease, modestly elevated risk of the stricturing CD phenotype, and significantly higher risk of small bowel disease [115]. Another study revealed that the presence of a single NOD2 mutation was associated with an 8% increase in the risk of a complicated CD course (stricturing or fistulizing) and a 41% increase in the risk among subjects owning two mutations. Furthermore, individuals with any NOD2 mutation presented a 58% elevated risk of needing surgery, whereas the risk of perianal disease remained unchanged. The authors of the study assumed that CD patients with two mutations of NOD2/CARD15, due to a high risk of a complicated course of the disease, may benefit from the early intensification of therapy [116]. On the contrary, some studies have not confirmed the association of NOD2/CARD15 variants with the stricturing course of CD [25,101,102]. It is still unclear whether the observed relationship between NOD2/CARD15 gene variants and stricturing CD is a real association, or only a reflection of a high proportion of CD patients who develop complications. A significant limitation of using gene variants as biomarkers is the fact that this does not take into account the impact of environmental factors on disease course, such as microbiome or nutrition.

Another group of interest as candidate biomarkers of stricturing CD are circulatory micro-RNAs (miR)—short noncoding RNA fragments regulating the gene expression in epigenetic mechanisms. Aberrant miRNA expression is related to the pathogenesis of fibrosis. Suppression of miR-29 has been linked to liver or renal fibrosis [117,118]. A study by Lewis et al. identified miR-19-3p to be a potential marker of the stenotic phenotype of CD. Patients with stricturing CD, compared to control CD patients, presented reduced serum concentrations of miR-19a-3p and miR-19b-3p (*p* = 0.007 and *p* = 0.008, respectively). The association between miR-19-3p and stenotic CD seemed to be independent of clinical factors, such as disease duration, disease activity, location, gender, or age. A 4-year patient follow-up supported this hypothesis [119]. Other variants of miR-19 have been also linked to fibrotic processes. A lower concentration of miR-19a-5p in the peripheral blood was found in interstitial lung fibrosis, as well as cardiac and liver fibrosis [120,121,122]. The usefulness of miRNA in IBD management was also confirmed in a prospective study conducted on 77 IBD patients. The authors stated that miR-320a blood levels were strongly correlated with the exacerbation of CD and reflected endoscopic and clinical disease activity, as well as reaching a response to treatment [123]. The limitations to the potential utility of miRNA as a biomarker include difficulties with isolation and purification.

## 4. Conclusions

Despite the increasing number of studies on the pathogenesis of fibrosis, our understanding of the patomechanisms of the stricturing CD phenotype and association between biomarkers and strictures remains limited. Currently, there is still a lack of clinically approved biomarkers of intestinal fibrostenosis. High hopes were raised for microbial biomarkers due to their specificity for gut microbiota and, thus, ileal fibrosis. However, ASCA antibodies or NOD2/CARD15-related markers seem to show a tendency towards a more severe CD course rather than being representative of the IBD stricturing phenotype. Promising results have been achieved according to other types of biomarkers—ECM compounds, such as COMP or growth factors, like HGFA. Despite a high correlation with the stricturing CD phenotype presented in previous studies and potentially easy obtainment, the main objection against their clinical usefulness may be a lack of specificity for ileal fibrosis. Most biomarkers derived from growth factors, cytokines, or ECM compounds have been already found to be associated with fibrosis in multiple other organs, which may be misleading in further studies. The development of imaging techniques has enabled GI tract stricture detection, however, distinguishing between inflammatory and fibrotic types of ileal stenosis is still ineffective. Non-invasive and easily obtained fecal biomarkers seem to have great potential, showing an eventual direction for fibrosis marker development. FC has a well-established position in IBD evaluation, as still no other valuable fecal biomarkers, specific for ileal fibrosis, have been recently found.

Expansion in drug development has led to better control of inflammation in IBD course, however, the available anti-inflammatory therapies still have little impact on the reduction in or reversibility of GI tract fibrosis, remaining a great medical challenge. The progression of intestinal fibrosis is partially independent of the inflammatory process and indicates an urgent need for the identification of reliable, noninvasive biomarkers, which could be useful in the management of IBD patients, especially those with CD. Further studies on the pathogenesis underlying the stricturing CD phenotype and its associated biomarkers may contribute to the optimalization of IBD patients’ management and better long-term outcomes. Advances may be hampered by a lack of validated endpoints, which would enable scientists to compare the results of clinical trials.

## Figures and Tables

**Table 1:** Potential biomarkers of fibrogenesis in inflammatory bowel disease.

Category	Biomarkers
Extracellular matrix proteins	Collagen I Collagen III Collagen IV Collagen degradation products (fragments of type I (C1M), III (PRO-C3, C3M), IV (PRO-C4, C4M, C4G), and VI (C6Ma3)) Fibronectin isoform ED-A Cartilage oligomeric matrix protein (COMP)
Growth factors	Transforming growth factor β (TGF-β) Hepatocyte growth factor activator (HGFA)
Cytokine antibodies	Anti TGF-β antibodies Anti interleukin 10 (IL-10) antibodies Anti granulocyte-macrophage colony-stimulating factor (GM-CSF) antibodies
Antimicrobial antibodies	Anti-Saccharomyces cerevisiae antibody (ASCA) Anti-zymogen granule membrane glycoprotein 2 (GP2) antibodies Anti-flagellins: A4-Fla2, anti Fla-X, anti-CBir1 Anti-Escherichia coli outer membrane porin C (anti-OmpC) Anti-CD associated bacterial sequence (I2) Cathelicidin (LL-37)
Genetic variants	Caspase activation recruitment domain (NOD2/CARD15) L-selectin (CD62L) Micro-RNA (miR-19a-3p, miR-19b-3p) Genetic variation of cytokines: IL-12B, IL 10 Tumor necrosis factor (TNF) ligand superfamily member 15
Histhopathology/Tissue based markers	Mast cell density TGF-β activated kinase 1 (TAK1) Ovarian cancer G-protein coupled receptor 1(OGR1) mRNA Cholesterol 25 hydroxylase (CH25H) mRNA
Other	Fecal calprotectin (FC) Fecal lactoferrin (FL) a2-Heremans-Schmid glycoprotein (AHSG/fetuin A) Elafin Mannan-binding lectin (MBL) C-reactive protein (CRP)

## Data Availability

No new data were created.
